# Herpes Simplex Virus (HSV) Pneumonitis as a Late Complication of COVID-19 Infection: A Unique Case Report

**DOI:** 10.7759/cureus.79228

**Published:** 2025-02-18

**Authors:** Shirisha Pasula, Rupalakshmi Vijayan

**Affiliations:** 1 Internal Medicine/Infectious Diseases, University of California, Fresno, USA; 2 Family Medicine, St. Elizabeth Mercy Boardman Hospital, Boardman, USA

**Keywords:** hemoptysis, hsv pneumonitis, immunocompetent adults, post-covid-19 complications, pulmonary infiltrates

## Abstract

This case report details a patient who presented with gradually worsening shortness of breath and hemoptysis over a year following her COVID-19 infection. Notably, the patient was otherwise immunocompetent. After a comprehensive evaluation, she was diagnosed with herpes simplex virus (HSV) pneumonitis. The patient was treated with oral acyclovir, which is the standard approach for managing HSV pneumonitis. This case highlights the potential for delayed HSV reactivation and the need for awareness of such complications in patients recovering from COVID-19.

## Introduction

The herpes simplex virus (HSV) belongs to the family of Herpesviridae. HSV is primarily transmitted through mucosal contact or through disrupted skin barriers. After the initial infection, the virus can remain dormant in neural ganglion and may reactivate under certain conditions, such as stress, illness, or immunosuppression [1]. The clinical presentation of HSV varies depending on the anatomic site involved and the host's immune response, with manifestations ranging from oral and genital skin lesions to more severe complications like encephalitis [1]. HSV pneumonitis can occur in immunocompromised and mechanically ventilated patients, but it is rare in immunocompetent individuals.

Recent studies have highlighted a notable association between COVID-19 and HSV reactivation, particularly HSV-1 [2]. The immune dysregulation and lymphopenia commonly observed in severe COVID-19 cases, along with the immunosuppressive treatments used in managing the infection, may predispose patients to reactivation of latent HSV. This phenomenon underscores the complexity of viral interactions in the context of COVID-19, as the heightened immune response to COVID-19 can inadvertently compromise defenses against other viral pathogens. Understanding this relationship is crucial for clinicians in managing potential co-infections and optimizing treatment strategies for affected patients.

## Case presentation

A 72-year-old female presented to the emergency room with hemoptysis following an aborted left shoulder surgery. During intubation at an outside facility, blood was noted in the trachea, leading to the termination of the procedure and her subsequent transfer for further evaluation. The patient’s medical history included a mild COVID-19 infection approximately one year prior, characterized by a dry cough and fatigue, which did not necessitate hospitalization or treatment.

Since the initial infection, the patient reported a persistent dry cough that developed into hemoptysis over the past two months. She also experienced progressively worsening shortness of breath, which made simple activities, such as climbing a flight of stairs, increasingly challenging for two months. The patient denied any fever, abdominal pain, oral ulcers, nausea, vomiting, or weight loss.

The patient had a past medical history significant for hypertension, diabetes mellitus, and left ventricular outlet obstruction. The surgical history included carotid artery stent placement seven years ago to treat significant carotid artery stenosis. The patient’s family history is notable for maternal hypertension and a paternal history of melanoma. The patient has a history of tobacco use, which she discontinued eight years ago, and denies any alcohol consumption or injection drug use.

Upon presentation, vital signs were stable: temperature 98.6°F, heart rate 70 beats per minute, respiratory rate 18 breaths per minute, and blood pressure 148/61 mm Hg, with oxygen saturation at 95% on room air. The oral examination revealed no mucosal lesions. Auscultation of the lungs demonstrated diffuse bilateral crackles, while cardiac examination identified a systolic murmur, which was consistent with left ventricular outlet obstruction. The abdomen was soft and non-tender upon palpation. The patient’s left arm was immobilized in a sling, and there was no evidence of pedal edema or skin lesions.

Laboratory tests are mentioned in Table 1. They indicated a normal white count with mild lymphopenia and normal procalcitonin.

**Table 1:** Laboratory results HIV, human immunodeficiency virus

Lab test	Result	Reference range and units
White blood cell count	7.6/µL	4.5-11.5/µL
Absolute lymphocyte count	0.6/µL (low)	1.5-4/µL
Hemoglobin	11.4 g/dL	11.5-15.5 g/dL
Platelet count	156/µL	130-450/µL
Creatinine	0.7 mg/dL	0.5-1 mg/dL
Procalcitonin	0.03 ng/mL	0-0.08 ng/mL
Serum Cryptococcal antigen	Negative	Negative
HIV screen	Negative	Negative
Tuberculosis spot test	Negative	Negative

The differential diagnosis encompasses a range of infectious and non-infectious etiologies. Infectious considerations include atypical pneumonia and opportunistic infections such as *Pneumocystis jirovecii* pneumonia or other fungal infections, particularly given a prior history of COVID-19 infection. Non-infectious etiologies include autoimmune diseases like vasculitis or interstitial lung disease. Further diagnostic evaluation, including imaging studies and potentially bronchoscopy, is warranted to elucidate the underlying cause of her clinical presentation.

**Figure 1:**
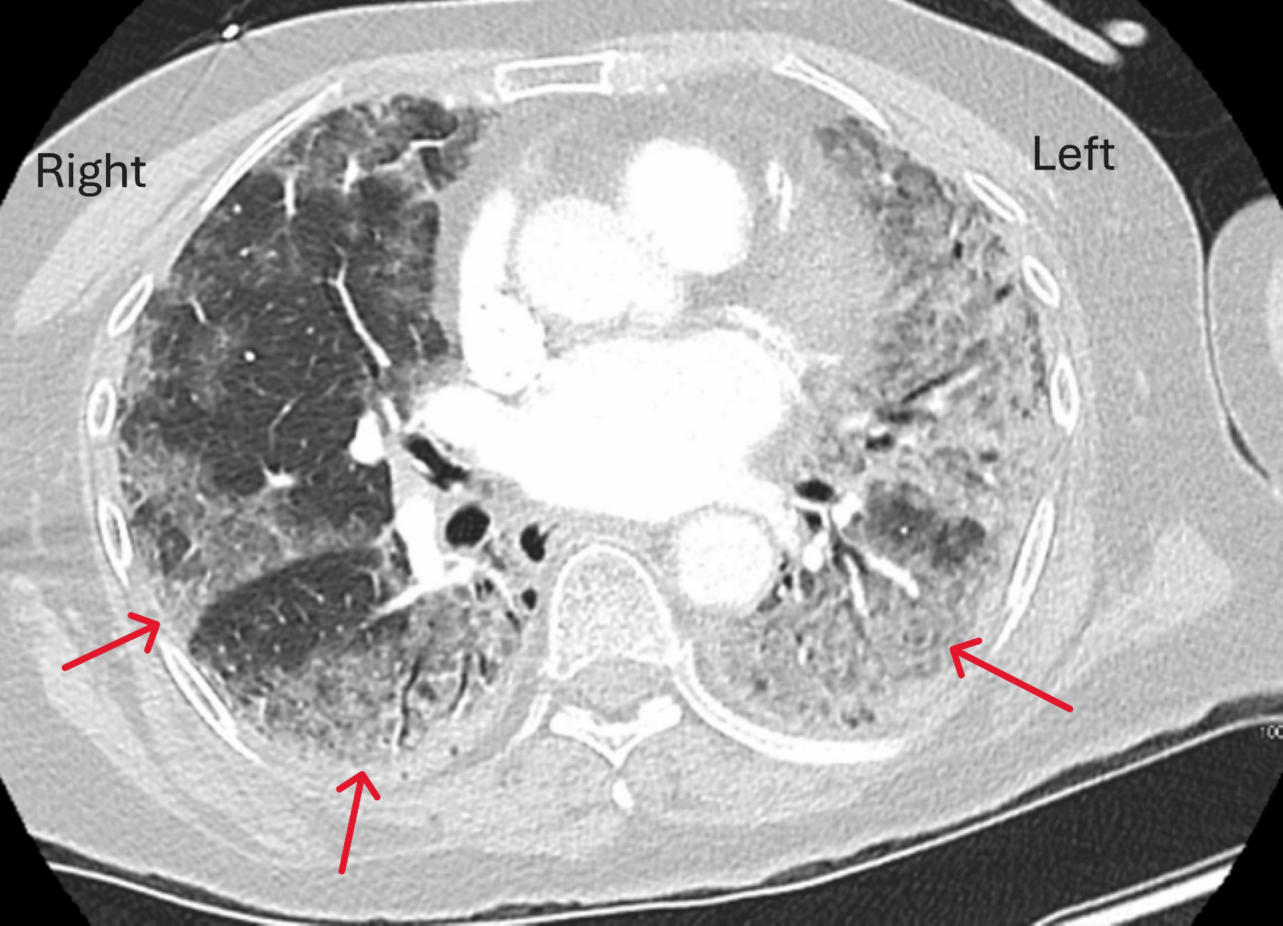
CT chest showing diffuse pulmonary infiltrates, with the entire left lung involved and patchy infiltrates in the right lung CT, computed tomography

**Figure 2:**
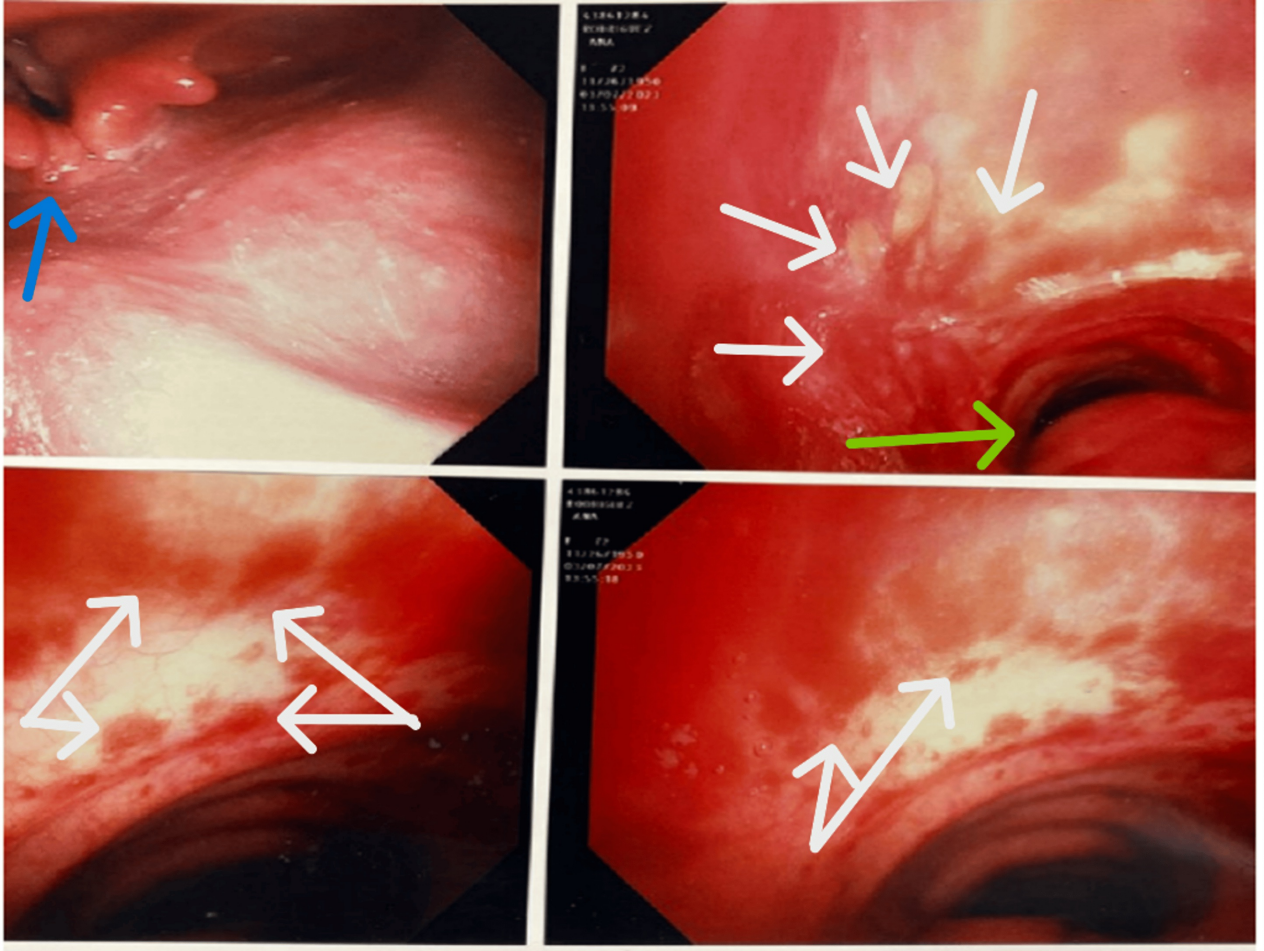
Bronchoscopy The blue arrow points to the vocal cords and the normal mucosa above them. The green arrow points to the trachea, and the white arrows indicate severe mucosal inflammation and bleeding in the trachea and bronchus.

Computed tomography (CT) imaging of the chest showed extensive bilateral pulmonary infiltrates (Figure 1). Initial investigations, including a respiratory viral panel using a nasopharyngeal swab, MRSA nasal screen, and urine antigen tests for *Streptococcus pneumoniae* and *Legionella pneumophila*, yielded negative results. The patient was treated with intravenous ceftriaxone 1 g daily and oral doxycycline 100 mg twice a day for five days, but there was no clinical improvement. Comprehensive autoimmune serologies, including antinuclear antibodies (ANA), anti-double-stranded DNA, and anti-glomerular basement membrane (GBM) antibodies, returned negative. Further infectious disease evaluation with serum beta-D-glucan, cryptococcal antigen, human immunodeficiency virus (HIV) screen, and tuberculosis spot test were also negative. On day three, a diagnostic bronchoscopy was performed, revealing friable mucosa and diffuse alveolar hemorrhage (Figure 2). Diffuse alveolar hemorrhage was considered in the differential and the treatment started with steroid therapy (intravenous methylprednisolone).

Bronchoalveolar lavage (BAL) culture revealed oral flora, while mycobacterial and fungal stains and cultures were negative. PJP stain on BAL was negative, but the BAL Aspergillus galactomannan level was noted to be 0.55 (reference range <0.5). Due to the risk of bleeding, lung biopsy was deemed high risk, and a repeat bronchoscopy on day six showed no improvement. Notably, the repeat BAL Aspergillus galactomannan level had come back at 1.76 (reference range <0.5), pulmonary aspergillosis was considered in the differential diagnosis. The patient was started on intravenous voriconazole; however, after two doses, the patient experienced visual hallucinations, leading to discontinuation of the medication, as the clinical presentation did not align with pulmonary aspergillosis. Further diagnostic workup with BAL Aspergillus PCR was negative, and serum Aspergillus galactomannan was also negative. At that point, the BAL Aspergillus galactomannan was thought to be falsely elevated. A second bronchoscopy's cytology revealed a single cell with changes suggestive of a viral cytopathic effect, consistent with HSV. On day 10, another bronchoscopy was performed, which demonstrated moderate improvement in alveolar hemorrhage. A bronchial mucosal biopsy was then performed, and HSV PCR was sent from the BAL. The patient was discharged on a tapering dose of steroids.

**Figure 3:**
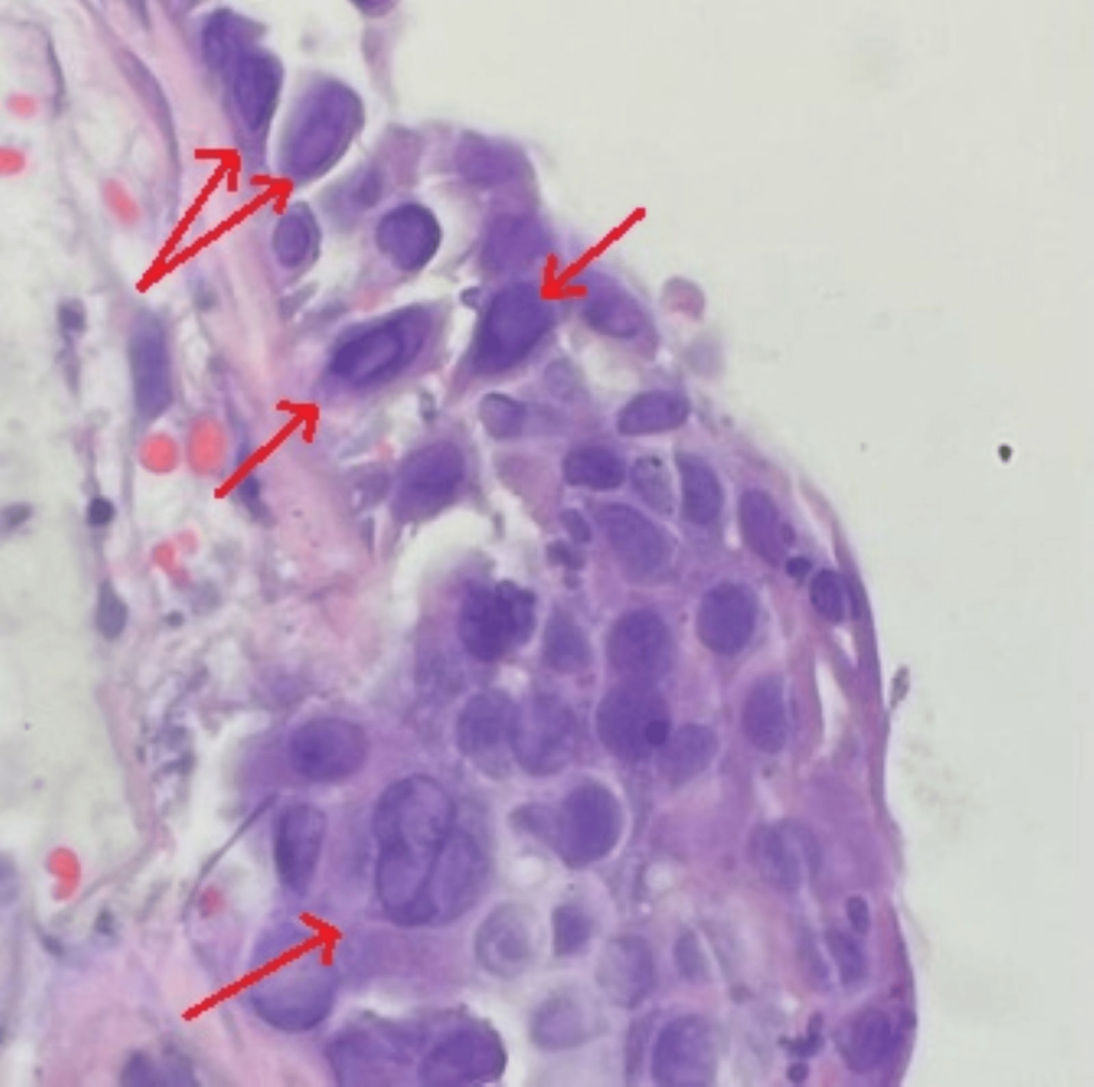
Bronchial mucosal pathology Denuded mucosa with HSV-like viral cytopathic effect (irregular nuclear membrane and intracytoplasmic inclusions shown by the arrow). HSV, herpes simplex virus

**Figure 4:**
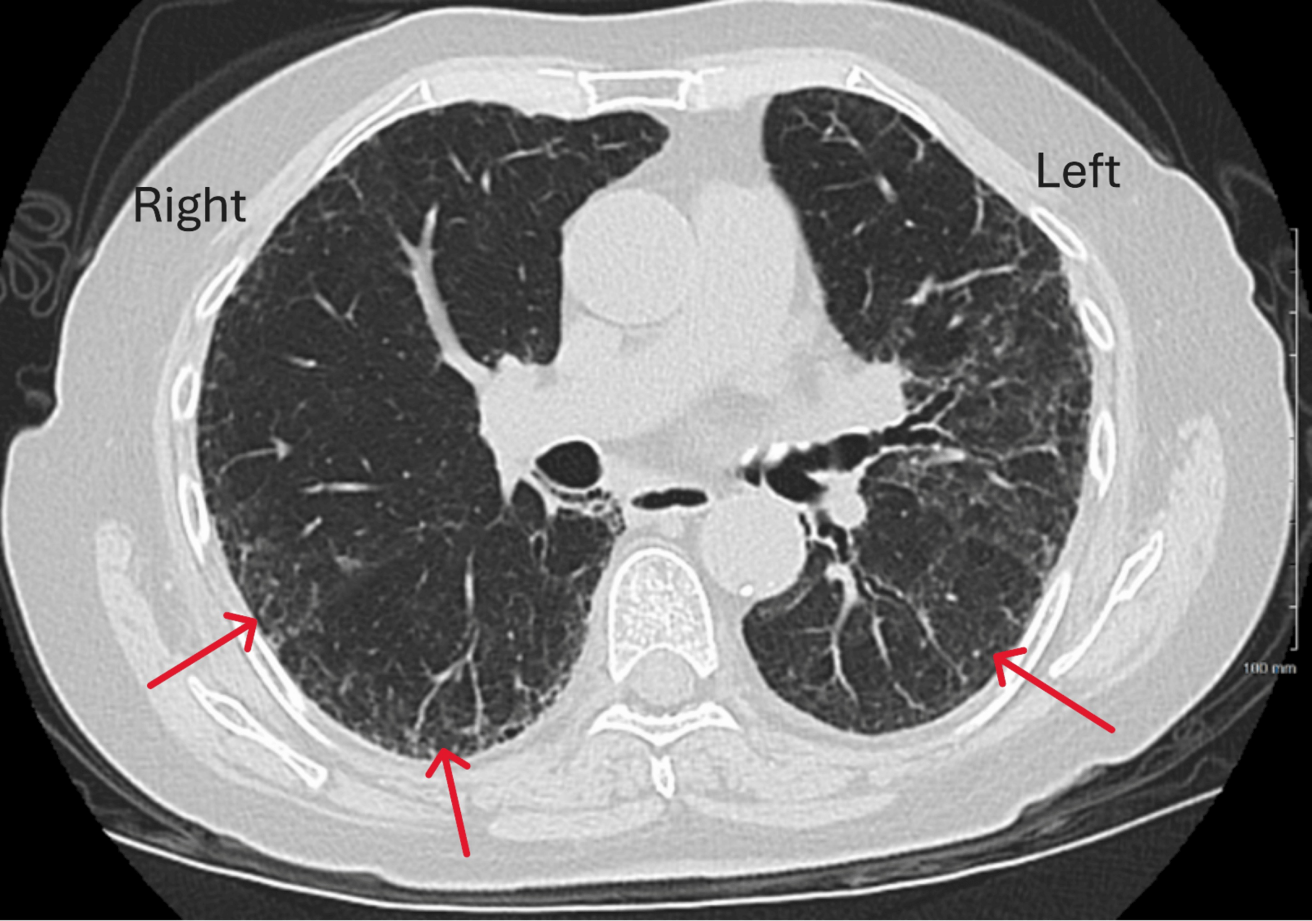
CT chest Significant improvement of pulmonary infiltrates in both lungs in comparison to Figure 1.

Following discharge, the mucosal biopsy revealed fragments of denuded bronchial mucosa with necro-inflammatory exudate and HSV-like cytopathic effects (Figure 3). BAL HSV PCR confirmed the presence of HSV-1, while BAL cytomegalovirus PCR was negative. A diagnosis of HSV 1 pneumonitis was established, and the patient was treated with oral acyclovir 400 mg three times a day for 14 days. The patient was followed up in the clinic three weeks after hospital discharge. She had a resolution of shortness of breath and hemoptysis, and a repeat CT chest was performed, showing significant improvement in pulmonary infiltrates (Figure 4).

## Discussion

A wide variety of presentations of HSV reactivation have been documented in patients with COVID-19, including HSV keratitis, meningitis, encephalitis, and even acute liver failure [3-5]. Especially, some affected individuals experienced only mild symptoms from COVID-19. HSV tracheobronchitis and pneumonitis are primarily observed in immuno-compromised and critically ill patients, with recent reports highlighting their occurrence in critically ill COVID-19 cases [6]. The reactivation of HSV has typically been noted to occur between six to 30 days following COVID-19 infection. To date, there appears to be no documented case of HSV reactivation leading to pneumonitis in an otherwise immunocompetent patient occurring over a year post-COVID-19 diagnosis.

The pathogenesis of HSV pneumonia is primarily believed to result from the aspiration of salivary secretions, which can affect the pharyngeal and tracheobronchial regions, ultimately leading to pneumonia [7]. In some cases, neurogenic or hematogenous spread has been reported, particularly in instances of disseminated herpes infection [8]. Nonetheless, the possibility of local pulmonary reactivation and primary hematogenous dissemination cannot be completely ruled out, highlighting the complexity of HSV's impact on the respiratory system and the need for further investigation into its various pathways of infection.

Diagnosing HSV tracheobronchitis or pneumonitis presents significant challenges, as there are no established diagnostic criteria. CT of the chest may reveal diffuse ground-glass opacities, peri-bronchial consolidations, or a combination of both [9]. A positive HSV culture or PCR from upper respiratory specimens may indicate subclinical shedding, particularly without clinical disease. However, the interpretation of HSV detection in lower respiratory tract specimens must be approached with caution, necessitating further evaluation through bronchoscopy. Clinically, HSV can induce endobronchial vesicles and mucositis, which can be visualized during bronchoscopy. Although BAL is a useful technique for confirming lower respiratory tract infections, bronchial washings often yield higher detection rates for HSV infections [10]. This distinction is important, as obtaining samples from bronchial washing can enhance diagnostic accuracy in suspected cases of HSV pneumonitis. Given the variable presentations and potential for misinterpretation of lower respiratory specimens, selecting the appropriate method for sampling is crucial in establishing a definitive diagnosis. In the case of endobronchial lesions, a biopsy of the affected tissue can provide valuable diagnostic information. In our patient, due to a high bleeding risk, tracheal or lung biopsy was considered too risky early on, which subsequently led to a delay in diagnosis.

The treatment of HSV infection depends on the site of involvement and the severity of the infection. Antiviral drugs to treat HSV infection include acyclovir, valacyclovir, and famciclovir [11]. Acyclovir is available in both oral and intravenous forms, while valacyclovir and famciclovir are available only as oral agents. The dosage of antiviral agents is according to the patient's renal function. The duration of treatment for mild to moderate infections is usually seven to 10 days. For severe infections and in immunocompromised patients, the duration of treatment depends on the clinical improvement. 

Meyer et al. reported that patients with HSV pneumonitis following COVID-19 infection experienced increased mortality and a higher risk of hospital-acquired pneumonia [12]. Acute respiratory distress syndrome (ARDS) secondary to COVID-19 pneumonia is highly associated with reactivation of HSV-1. The presence of HSV-1 in the lower airways has been correlated with poorer prognostic outcomes and significantly increased mortality [13]. The prognosis for these patients, along with the potential benefits of prophylactic measures in those with COVID-19, warrants further investigation to better understand optimal management strategies. Continued research is essential to elucidate the relationship between HSV reactivation and COVID-19 outcomes and determine effective preventive interventions. 

BAL Aspergillus galactomannan is a criterion in diagnosing pulmonary invasive aspergillosis according to the European Organization for Research and Treatment of Cancer/Mycoses Study Group (EORT-MSG) consensus criteria [14]. Most of the studies on galactomannan assay were conducted in patients with hematological malignancy or transplant patients [14]. False positive galactomannan results can occur in patients receiving certain medications, such as piperacillin-tazobactam, as well as in those with bacterial infections like *Pseudomonas aeruginosa*, or other fungal infections and colonization in the respiratory tract. In one study by Farmakiotis et al. there were 42% false positive BAL galactomannan in patients with hematological malignancy or transplant patients [14]. Using additional testing like BAL fungal cultures, Aspergillus PCR and serum Aspergillus galactomannan will be useful in ambiguous situations. Our patient did not have a clinical picture consistent with pulmonary aspergillosis and had negative BAL Aspergillus PCR as well as negative serum Aspergillus galactomannan leading to the belief that BAL galactomannan was a false positive.

## Conclusions

This is a report of an unusual case of HSV pneumonitis presenting as hemoptysis, particularly in a delayed post-COVID-19 recovery setting, highlighting the importance of considering atypical etiologies for respiratory symptoms. A high index of suspicion is necessary even after the acute viral phase has passed, as the delayed diagnosis can result in secondary infections going unrecognized. In this patient, the high risk of bleeding posed a significant challenge in obtaining a tracheal mucosal biopsy or lung biopsy early on, which led to a delay in diagnosis. This case also emphasizes the importance of long-term monitoring, at least for a year, in patients recovering from COVID-19 pneumonia, as they are at risk for rare but serious complications. More research is needed to better understand the interaction between COVID-19 and opportunistic viral infections like HSV, the prevalence of HSV infections post-COVID-19, and to establish optimal diagnostic and treatment strategies.
